# Editorial: Immunology and Immunotherapy of Head and Neck Cancer

**DOI:** 10.3389/fonc.2021.815763

**Published:** 2021-12-06

**Authors:** Niki Gavrielatou, Panagiota Economopoulou, Ioannis Kotsantis, Amanda Psyrri

**Affiliations:** ^1^ Department of Pathology, Yale University School of Medicine, New Haven, CT, United States; ^2^ Section of Medical Oncology, Department of Internal Medicine, Faculty of Medicine, National and Kapodistrian University of Athens, Attikon University Hospital, Athens, Greece

**Keywords:** head and neck cancer, immunotherapy, biomarkers, PD-1/PD-L1, HPV

Immunotherapy targeting programmed death-1/programmed death ligand-1 (PD-1/PD-L1) has been established as the standard of care in the first line of treatment for recurrent metastatic (R/M) HNC, either in combination with chemotherapy or as monotherapy for PD-L1 positive tumors, as was meticulously reviewed in the current topic by Hsieh et al. Nonetheless, results from several clinical studies have shown limited efficacy of immune checkpoint inhibition in earlier disease stages. For locally advanced disease immunotherapy has been explored in two distinct frameworks; 1. in combination with chemoradiotherapy for treatment intensification in high risk cases, or 2. as an alternative resource, aiming at the de-intensification of high-toxicity chemoradiotherapy regimens, in low-risk cases. Immunotherapy in the neoadjuvant setting is also investigated in several clinical trials, primarily targeting high risk/HPV negative cases, either as single agent or in chemotherapy combinations. Although induction immunotherapy exhibited an acceptable safety profile in most clinical trials, it has not been studied in randomized studies and thus has not received approval in the neoadjuvant setting. Additionally, for locally advanced disease, Saddawi-Konefka et al. also provide a comprehensive summary on the current immunotherapy-related preclinical and clinical data. As the addition of PD-L1 inhibitor to cisplatin chemoradiotherapy has failed to demonstrate advantage over cisplatin chemoradiotherapy alone in randomized III trials, the authors highlight the importance of optimizing therapeutic sequence as a potential way to maximize benefit from immune checkpoint inhibition. Moreover, in an interesting case report included in the present Research Topic, Nie et al. propose an alternative treatment approach for locally recurrent disease after surgery, which involves the combination of an PD-1/PD-L1 inhibitor with an EGFR targeted agent, followed by radiotherapy. The combined regimen led to nearly complete tumor regression and consequent improvement in a PD-L1 positive case, where prognosis with conventional therapeutic options would be otherwise dismal. However, it should be noted that the observed positive outcome was accompanied by a grade IV autoimmune adverse event, indicating the need to perform a cost-benefit evaluation of the proposed schema in larger patient cohorts.

Biomarker analysis performed as part of clinical trials, which led to the introduction of immunotherapy in R/M HNC management, have shown a direct correlation of drug efficacy with PD-L1 protein expression, which resulted in PD-L1 becoming the only clinically approved predictive biomarker of immunotherapy response in this type of cancer. However, as a significant proportion of PD-L1 positive cases experience no clinical benefit, a large body of research has focused on the tumor microenvironment (TME) in search of diverse predictive biomarkers with potential clinical applicability. In their article on the present Research Topic, Zhao et al. identified the immune-inhibitory functions of IL-21 in HNC TME and described its negative prognostic effect. Using patient and healthy-donor tissue samples, they found association of increased IL21+CD4+ T helper cells’ presence in stroma with advanced disease stage and poor survival outcomes. Additionally, IL-21 prompted cell polarization towards the regulatory (Treg) phenotype and induced Treg generation and expansion utilizing a PD-1/PD-L1 interaction-dependent mechanism. Interestingly, IL21- stimulated Tregs specifically, appeared to suppress immunity against tumor associated antigens, an effect that was reversed by dual IL-21 and PD-1 inhibition. The above finding provides insight on a possible mechanism of resistance to immunotherapy in HNC and at the same time sets the groundwork for further validation of IL-21 as a potential predictive biomarker for response to PD1 checkpoint inhibitors.

A different approach in biomarker development involves gene expression analysis and molecular characterization of head and neck tumors. Accordingly, RNA analysis on PD-L1 differential expression among HNC patients by Wu et al., revealed that increased baseline PD-L1 expression correlated with poor prognosis and the “PD-L1 high” subgroup exhibited upregulated tumor-associated macrophage gene expression, as well as increased expression of epithelial mesenchymal transition-related genes. The authors also conclude that the same patient category demonstrated improved response both to immunotherapy and chemotherapy agents. Additionally, Wang et al. proposed a 21 long-noncoding- RNA-pair immune related signature, derived from TCGA data, as both a prognostic tool and an immunotherapy predictive biomarker. It should be noted that although gene signatures often exhibit promising predictive value, they are hard to validate in large real-world cohorts and as their identification requires costly assays, they represent controversial candidates for clinical implementation as companion diagnostic tests.

Moreover, HPV positivity is known to characterize a biologically distinct sub-category of head and neck tumors, with favorable prognosis and abundant immune infiltration. As findings from previous studies have failed to draw definite conclusions on the potential implication of HPV status in immunotherapy outcomes, Xu et al. attempt to shed further light on the Research Topic by analyzing combined study data. In their meta-analysis of 814 patients with known HPV status, they observed a clear association of HPV positivity with increased objective response rate and overall survival in anti-PD-1/PD-L1 treated cases and the same favorable trend was observed for disease control rate and progression- free survival. The above findings suggest that HPV- related pre-existing tumor immunogenicity enhances the therapeutic effect of immune checkpoint inhibition.

In conclusion, the present Research Topic has accumulated impactful articles aiming at the compilation of current knowledge on immunotherapy for HNC, as well as the illustration of novel findings on immunotherapy biomarkers.

## Author Contributions

All authors listed have made a substantial, direct, and intellectual contribution to the work and approved it for publication.

## Conflict of Interest

The authors declare that the research was conducted in the absence of any commercial or financial relationships that could be construed as a potential conflict of interest.

## Publisher’s Note

All claims expressed in this article are solely those of the authors and do not necessarily represent those of their affiliated organizations, or those of the publisher, the editors and the reviewers. Any product that may be evaluated in this article, or claim that may be made by its manufacturer, is not guaranteed or endorsed by the publisher.

